# 1857. Why do antibiotic become resistant in hospitalized patients? Evidence from tertiary healthcare facilities in Bangladesh

**DOI:** 10.1093/ofid/ofad500.1685

**Published:** 2023-11-27

**Authors:** Shariful Amin Sumon, A B M Alauddin Chowdhury, Sabrina Sharmin, Md Golam Dostogir Harun

**Affiliations:** icddr,b, Mohakhali, Dhaka, Bangladesh; Daffodil International University, Dhaka, Dhaka, Bangladesh; Bangabandhu Sheikh Mujib Medical University, Dhaka, Dhaka, Bangladesh; icddrb, Dhaka, Dhaka, Bangladesh

## Abstract

**Background:**

Antibiotic resistance among hospitalized patients is a growing concern due to irrational antibiotic consumption. Exploring the dynamics of antibiotic consumption including healthcare-seeking behavior is indispensable to minimizing the antibiotic resistance burden. This study explored the pattern of antibiotic consumption and healthcare-seeking behavior of patients before being admitted to tertiary hospitals in Bangladesh.

**Methods:**

Between August and September 2022, we conducted this cross-sectional study in four tertiary care ( 2 public and 2 private) hospitals. A total of 943 admitted patients were randomly enrolled and face-to-face interviews were conducted to obtain data on antibiotic usage history, healthcare-seeking patterns, and duration of medical consultation. Descriptive statistics were used for the analysis and interpretation.

**Results:**

Prior to hospitalization, 32.8% (95%CI: 30.5, 35.1) of patients had antibiotic consumption history within the previous two months without lab confirmation. About one-quarter (25.6%) of the participants sought antibiotic treatment from unregistered physicians. Patients admitted to private hospitals were found to be higher to seek treatment from unregistered sources compared to public hospitals (37.8% vs. 13.4%, p-value: 0.001). Less than two-thirds (63.8%) of patients completed their entire course of antibiotics before admission to the hospitals. The average duration between symptom onset and medical consultation was 5.6 (SD 1.3) days before hospitalization. In terms of place for healthcare seeking, the majority of participants (52.0%) visited government hospitals, followed by private hospitals (22.6%), and pharmacies (20.1%). Self-medication was found in 5.3% of participants before being admitted to the hospital. However, 72.2% of patients were prescribed antibiotics after admission and only 5.3% had a laboratory diagnosis before receiving antibiotics.

**Conclusion:**

Hospitalized patients were found to consume antibiotics more frequently before and after admission. Furthermore, healthcare seeking from unregistered sources, including delayed and incomplete treatment could hindrance to combating against antibiotic resistance among admitted patients.

**Disclosures:**

**All Authors**: No reported disclosures

